# National treatment patterns in patients presenting with Stage IVC head and neck cancer: analysis of the National Cancer Database

**DOI:** 10.1002/cam4.546

**Published:** 2015-10-16

**Authors:** Zachary G. Schwam, Barbara Burtness, Wendell G. Yarbrough, Saral Mehra, Zain Husain, Benjamin L. Judson

**Affiliations:** ^1^ Department of SurgerySection of OtolaryngologyYale University School of MedicineNew HavenConnecticut; ^2^ Department of Internal MedicineSection of Medical OncologyYale University School of MedicineNew HavenConnecticut; ^3^ Department of Therapeutic RadiologyYale University School of MedicineNew HavenConnecticut

**Keywords:** Clinical trials as topic, head and neck cancer, neoplasm metastases, outcomes assessments, palliative care

## Abstract

Head and neck cancer patients presenting with distant metastases are generally considered incurable. Treatment patterns and survival by primary disease site and therapy have not been described. Retrospective cohort analysis of 2525 patients in the National Cancer Database (2003–2006). Kaplan–Meier and Cox proportional hazards analyses were performed. Combined locoregional and systemic therapy was the most common treatment regimen (39.2%), followed by no treatment (23.9%), locoregional (19.0%), and systemic treatment (17.8%). Multivariate analysis demonstrated decreased survival was associated with age 65–79 years hazard ratio [HR] 1.43, 95% confidence interval [CI] 1.14–1.80), Medicaid/uninsured status (HR 1.27, 95% CI 1.13–1.42), Medicare/other government insurance (HR 1.21, 95% 1.07–1.38), treatment at a nonacademic/research program (HR 1.17, 95% CI 1.07–1.27), and Charlson comorbidity score of 1 (HR 1.33, 95% 1.19–1.48). Compared to systemic therapy alone, receiving locoregional and systemic therapy was associated with decreased risk of death (HR 0.73, 95% CI 0.65–0.83). Only 14.6% and 0.6% of patients were recorded as receiving palliative therapy or being enrolled in a clinical trial, respectively. Significant treatment diversity exists in distantly metastatic head and neck cancer. Those who received combination locoregional and systemic therapy were more likely to have improved overall survival, but important factors in treatment selection are unknown. A small proportion of patients was found to receive either palliative therapy or was enrolled in a clinical trial, although these data likely underestimate the true proportions.

## Introduction

Approximately two‐thirds of patients diagnosed with head and neck cancer present with advanced disease 1, and up to 17% have been reported to present with distant metastases 1, 2, 3. Despite advances in diagnostic methods and imaging, the proportion of head and neck cancer patients presenting with Stage IV disease has remained stable since 1990 4. Large tumors of the hypopharynx, oropharynx, and oral cavity have the highest rates of distant metastasis, with the lungs, bone, and liver being the most common sites of spread 5, 6.

Patients with distant metastatic disease (Stage IVC [any T, any N, M1]) are generally considered incurable, with the focus of treatment being palliation, prolonging survival, and optimizing quality of life 7. There is a strong element of diversity in the choice of treatment for late‐stage head and neck cancer 4, 8, especially for those patients with distant metastases. Current national practice guidelines suggest enrollment in a clinical trial, locoregional treatment, standard systemic therapy, or best supportive care, depending on performance status 9.

There is a scarcity of data with respect to national treatment patterns for Stage IVC head and neck cancer patients, and how treatment may affect survival in the setting of patient demographic and clinical factors. Successful salvage of patients with oligometastatic p16‐positive disease has been recently described 10, 11, but it remains unknown whether curative therapy can be undertaken for patients with synchronous disease. The goals of this analysis are to describe national treatment patterns by primary site, and to investigate their role in overall survival.

## Methods

### Data source

A retrospective analysis of the National Cancer Database (NCDB) was performed for patients diagnosed with Stage IVC head and neck cancers between years 2003 and 2006. The NCDB was established in 1989 and is a joint project of the Commission on Cancer (CoC) of the American College of Surgeons and the American Cancer Society 12. The NCDB contains data for approximately 70% of cancers diagnosed in the United States, drawing from over 1500 CoC‐approved hospital‐based registries. The data reporting process to the NCDB is highly standardized, the rules of which are outlined in the CoC data acquisition manuals 13.

### Selection criteria

Primary sites were determined by International Classification of Disease in Oncology (ICD‐O‐3) topography codes and included the oral cavity (C00.0‐00.6, 00.8, 00.9, 02.0‐02.3, 02.8, 02.9, 03.0, 03.1, 03.9, 04.0, 04.1, 04.8, 04.9, 05.0, 05.8‐06.2, 06.8, 06.9), oropharynx (C01.9, 02.4, 05.1, 05.2, 09.0‐09.1, 09.8, 09.9, 10.0‐10.4, 10.8, 10.9), nasopharynx (C11.0‐11.3, 11.8, 11.9), hypopharynx (C12.9, 13.0, 13.1, 13.2, 13.8, 13.9), as well as the glottic‐ (C32.0, 32.2, 32.3, 32.8, 32.9), and supraglottic larynx (C32.1) 14. Staging schema reflects the American Joint Committee on Cancer (AJCC) staging system, 6th edition 15.

We initially identified 412,825 patients with all stages of oral cavity, pharyngeal, and laryngeal cancer who had complete classification, staging, and tumor primary site data. We used this larger cohort to calculate the proportion of patients presenting with distant metastases by primary site. We then selected for all patients initially presenting with distant metastatic disease, and identified 11,637 patients. Patients with prior cancers or multiple primary cancers at the time of diagnosis were excluded from our analysis. Diagnostic confirmation was done histologically or cytologically in 97.7% of cases, and radiographically in 1.5% of cases. The remainder of the cohort had an unknown confirmation process. Cases with incomplete data for the following variables were then excluded from our analysis: patient age, gender, race, Hispanic origin, insurance status, income, education, proximity to a metropolitan area, facility location, facility type, Charlson score (collected from 2003 onward), tumor primary site, treatment‐related variables such as receipt of chemotherapy, radiation, surgery, participation in a clinical trial, receipt of palliative care, and follow‐up information including last known contact/death and vital status (collected until 2006). It was not possible to evaluate metastatic tumor burden (i.e., number and site of metastases) or sequence of therapy due to missing data on time to treatment initiation.

### Definition of patient variables

Variable definitions are consistent with those found in the NCDB data dictionary 16, except as noted here. Information regarding Hispanic origin was incorporated into the variable for race. Insurance status was characterized as private, uninsured/Medicaid, and Medicare/other government. Income and education data are from the 2000 U.S Census 17. Patients were stratified into those residing in a zip code with median household income <$30,000 per year (lowest quartile) and ≥$30,000 per year. Education reflects the percentage of the population in the patient's zip code having received a high school diploma. Outcomes included ≤71% (lowest quartile) and >71% with a high school diploma. Patients' proximity to a metropolitan area is derived from continuum codes set forth by the U.S. Department of Agriculture 18, and was subdivided into in, adjacent to, or nonadjacent to a metropolitan area, regardless of population size. Facility type reflects the category classification assigned by the Committee on Cancer Accreditation program 16 and was divided into Academic/Research Cancer Programs (ARPs) and non‐ARPs 19. Facility locations were originally coded as divisions of the U.S. census, but were recoded to reflect the census regions of Northeast, Midwest, South, and West 17. The Charlson score is a widely used method for classifying comorbidities and does not include the patient's cancer 20, 21. The scores are derived from International Classification of Disease (ICD) codes and have been truncated by the NCDB into 0, 1, or ≥2.

### Definition of treatment variables

Patients were coded as having received locoregional, systemic, locoregional and systemic, or no therapy as the first course of treatment. Systemic therapy included either single‐agent, multi‐agent, or unspecified systemic anti‐cancer therapy, whereas locoregional therapy included surgical resection of the primary, external beam radiation alone, or external beam radiation with isotopes or implants. It was not possible to determine the order of therapy in those receiving both locoregional and systemic treatment due to missing data. Distant‐site surgery and radiation outside of the head and neck region were not included as treatment with therapeutic intent. Patients in institution‐based or double‐blind clinical trials as part of the first course of treatment (within the variable “other treatment”) were coded as participating in a clinical trial; data for this variable have been collected since as early as 1998 and include other outcomes such as “none,” “unproven (administered by nonmedical personnel),” “refusal,” and “unknown.” Only those coded as being enrolled in an institution‐based or double‐blind trial were coded as positive for this variable, and patients with unknown status were not included in our cohort. While other clinical trial types exist, these are the only types recorded in the NCDB, and no distinction was made as to whether or not these trials were interventional in nature. Data regarding palliative therapies are collected by the facility registrar, and include surgery, radiation, chemotherapy, and pain management. Supportive care is not included in the definition of this variable. Treatment was coded as palliative if the primary intent was to control symptoms, alleviate pain, or increase comfort 16. If the palliative treatment was found to affect the primary tumor or metastases, it was also coded as part of the first course of treatment.

### Statistical analysis

Statistical analyses were performed using SPSS version 22.0.0 for Mac (Chicago, IL). Standard descriptive statistics were used to summarize demographic and disease‐related information. Kaplan–Meier log‐rank tests were performed for univariate survival analyses, and variables with *P* ≤ 0.10 were included in multivariable Cox proportional hazards regression. All tests were two‐sided and the final threshold for significance was set at *P* ≤ 0.05. Hazard ratios (HRs) and 95% confidence intervals (CIs) were reported. Corrections were not made for multiple comparisons testing.

As the NCDB is a de‐identified dataset, this study was granted exemption by the Yale University Human Investigation Committee.

## Results

Upon initially examining 412,825 head and neck cancer patients with complete staging information in the NCDB, we found the most common primary sites presenting with distant metastases to be the nasopharynx (7.7%) and hypopharynx (6.0%) (Table 1). We then identified 2525 patients with Stage IVC head and neck cancer, on which the rest of our analysis is based. Median follow‐up time was 8.1 months (range 0.0–117.3 months). The majority of patients with Stage IVC disease were male, between 45 and 79 years, White, and had Medicare/other government insurance (Table 2). Most patients were free of comorbid conditions and received treatment in non‐ARPs. The most common primary sites in our cohort of 2525 Stage IVC patients were the oropharynx, supraglottic larynx, and oral cavity (Table 1).

**Table 1:** Tumor primary site and percent of tumors presenting with distant metastases

Primary site	% (*N* = 2525)	Percentage of disease site cohort with distant metastases^a^
Oral cavity	15.0 (378)	1.4
Oropharynx	35.3 (892)	3.2
Nasopharynx	9.7 (246)	7.7
Hypopharynx	13.3 (335)	6.0
Glottic larynx	11.6 (292)	1.5
Supraglottic larynx	15.1 (382)	3.5
Total	100.0	2.8

a Represents the proportion of patients with tumors of each primary site presenting with distant metastases (*N* = 412,825 for all sites combined).

**Table 2:** Patient baseline characteristics and facility data

	% (*N* = 2525)
Sex	
Male	75.8 (1915)
Female	24.2 (610)
Age (years)	
18–44	5.4 (136)
45–64	50.1 (1265)
65–79	35.8 (904)
≥80	8.7 (220)
Race	
White	70.1 (1771)
Black	19.8 (501)
Hispanic	5.6 (142)
Asian	3.7 (94)
Other	0.7 (17)
Insurance	
Private	27.1 (684)
Uninsured/medicaid	27.2 (686)
Medicare/other government	45.7 (1155)
Income	
≥$30,000/year	78.0 (1969)
<$30,000/year	22.0 (556)
Education	
>71% with HSD	74.0 (1869)
≤71% with HSD	26.0 (656)
Proximity to metro area	
In metro area	82.5 (2084)
Adjacent to metro area	12.6 (317)
Not adjacent to metro area	4.9 (124)
Charlson score	
0	77.3 (1952)
1	17.6 (445)
≥2	5.1 (128)
Facility location	
Northeast	21.0 (531)
Midwest	23.5 (593)
South	40.2 (1015)
West	15.3 (386)
Facility type	
ARP	37.3 (941)
Non‐ARP	62.7 (1584)

HSD, high school diploma; ARP, academic/research program.

A combination of locoregional and systemic therapy was the most popular treatment regimen (39.2%), regardless of primary site (Table 3). Of those receiving combination therapy, 95.4% received radiation, and 12.3% underwent surgery. Systemic therapy alone was most commonly administered to patients with naso‐ and hypopharyngeal primaries, whereas locoregional treatment alone was most commonly given to patients with oral cavity and laryngeal tumors. Those with lesions of the glottic larynx and oral cavity received no treatment in 28.8% and 28.0% of cases, respectively. Patients with T4 lesions were more likely to receive no treatment or systemic therapy only, whereas patients with *N* ≥ 2 nodal disease were more likely to receive systemic ± locoregional therapy. Patients aged ≥65 years were less likely than those aged <65 years to receive combination locoregional and systemic therapy (30.6% vs. 46.1%, respectively, *P* < 0.001), as were patients with a Charlson score of ≥1 compared to 0 (33.3% vs. 40.9%, respectively, *P* < 0.001). There was no significant difference between ARPs and non‐ARPs in the scope of care.

**Table 3:** First course of treatment by tumor primary site, staging classification, and Charlson score

Primary site	None (%)	LR only (%)	Systemic only (%)	Systemic + LR (%)	*P*	Palliative care (%)	*P*
Oral cavity	28.0	30.4	14.8	26.7	<0.001	11.9	0.616
Oropharynx	21.9	15.8	18.4	43.9		15.4	
Nasopharynx	18.7	10.6	24.4	46.3		14.2	
Hypopharynx	26.0	16.1	21.8	36.1		16.4	
Glottic larynx	28.8	21.9	14.7	34.6		14.4	
Supraglottic larynx	22.5	21.2	14.1	42.1		14.4	
T‐classification							
1	19.0	23.4	16.8	40.8	0.039	10.9	0.021
2	18.9	20.2	16.6	44.3		12.0	
3	18.9	18.6	16.5	46.0		14.4	
4	25.1	19.2	17.8	37.9		17.3	
N‐classification							
0	26.0	27.9	13.4	32.7	<0.001	12.9	0.017
1	26.5	21.4	16.1	36.0		11.6	
2	18.9	16.9	19.9	44.3		15.8	
3	17.9	15.6	17.3	49.1		19.4	
Charlson score							
0	22.6	18.3	18.2	40.9	0.001	14.5	0.931
1	27.2	22.7	14.8	35.3		14.8	
≥2	32.8	18.0	22.7	26.6		15.6	
Overall	23.9	19.0	17.8	39.2	–	14.6	–

Only 14 patients (0.6%) were coded as being in a clinical trial, and 14.6% of patients received palliative treatment of some kind. Patients receiving palliative care were more likely to have nonprivate insurance (79.1% vs. 71.8%, *P* < 0.001) and T ≥ 3 lesions (75.7% vs. 67.9%, *P* = 0.006). Charlson score, facility type, primary site, and income were not significantly different between the two groups. When radiation was administered with palliative intent, the median dose was 39.6 Gy and the median treatment duration was 31.0 days, compared to 50.0 Gy and 50.0 days for those receiving radiation with therapeutic intent. Unfortunately, it was not possible to determine the timing of palliative therapy relative to other treatments.

**Figure 1:**
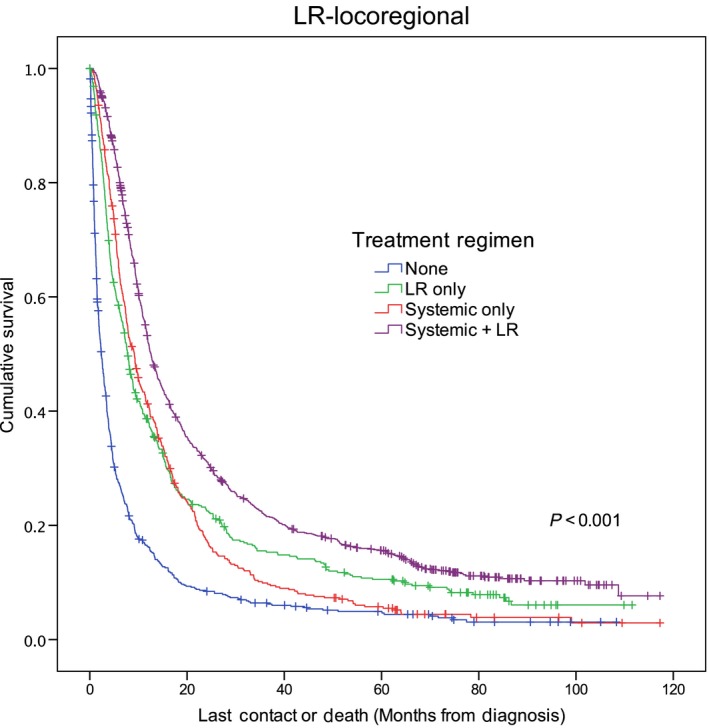
Kaplan–Meier overall survival curve stratified by treatment regimen. Hatches indicate censored data. LR, locoregional.

**Figure 2:**
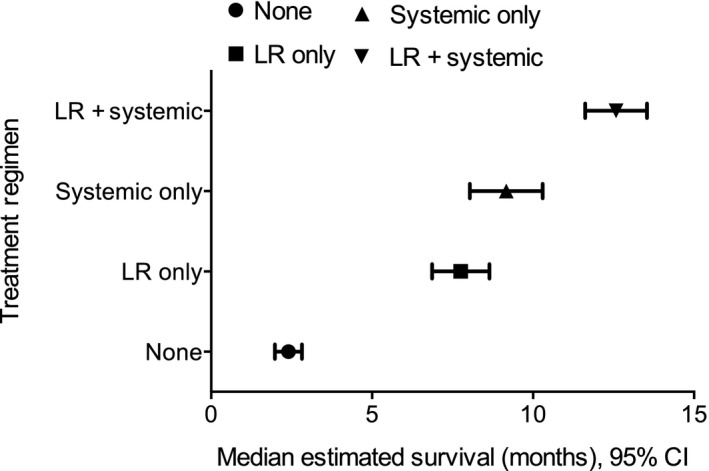
Median estimated survival time by treatment regimen. *P* < 0.001. LR, locoregional.

In univariate survival analysis, patients receiving combination locoregional and systemic therapy had the highest estimated median survival at 12.6 months, whereas those receiving no treatment had the lowest estimated median survival at 2.4 months (*P* < 0.001) (Figs. 1 and 2). Multivariable survival analysis demonstrated patient age ≥65 years and being uninsured or having government insurance to be associated with compromised overall survival (Table 4). Similarly, having a Charlson score of 1 or ≥2, receiving palliative therapy, and being enrolled in a clinical trial were associated with an increased risk of death. Patients with oropharyngeal primaries, when compared with those with primary tumors of the oral cavity, had improved overall survival. When compared with receiving systemic therapy alone, receiving combination locoregional and systemic therapy was associated with improved overall survival, while receiving no therapy was associated with mortality.

**Table 4:** Risk factors for mortality in multivariate survival analysis

	HR	95% CI	*P*
Sex			
Male (Ref)	1.00		
Female	0.92	0.84–1.02	0.110
Age (years)			
18–44 (Ref)	1.00		
45–64	1.22	0.99–1.50	0.066
65–79	1.43	1.14–1.80	0.002
≥80	1.80	1.38–2.34	<0.001
Race			
White (Ref)	1.00		
Black	1.06	0.95–1.19	0.315
Hispanic	0.89	0.73–1.08	0.230
Asian	0.86	0.66–1.11	0.235
Other	0.66	0.38–1.16	0.147
Insurance			
Private (Ref)	1.00		
Uninsured/medicaid	1.27	1.13–1.42	<0.001
Medicare/other govt	1.21	1.07–1.38	0.004
Income			
≥$30,000/year (Ref)	1.00		
<$30,000/year	1.01	0.89–1.14	0.930
Education			
>71% with HSD (Ref)	1.00		
≤71% with HSD	0.99	0.88–1.12	0.903
Proximity to metro area			
In metro area (Ref)	1.00		
Adjacent to metro area	0.97	0.85–1.10	0.586
Not adjacent to metro area	1.21	0.99–1.47	0.060
Facility location			
Northeast (Ref)	1.00		
Midwest	1.11	0.98–1.26	0.118
South	1.00	0.90–1.13	0.906
West	0.95	0.82–1.11	0.525
Facility type			
ARP (Ref)	1.00		
Non‐ARP	1.17	1.07–1.27	0.001
Charlson score			
0 (Ref)	1.00		
1	1.33	1.19–1.48	<0.001
≥2	1.57	1.30–1.89	<0.001
Primary site			
Oral cavity (Ref)	1.00		
Oropharynx	0.80	0.71–0.91	0.001
Nasopharynx	0.85	0.71–1.02	0.074
Hypopharynx	1.00	0.85–1.17	0.987
Glottic larynx	0.85	0.73–1.00	0.056
Supraglottic larynx	0.81	0.70–0.94	0.005
Treatment			
Systemic only (Ref)	1.00		
LR only	0.89	0.77–1.02	0.087
Systemic + LR	0.73	0.65–0.83	<0.001
None	2.03	1.78–2.31	<0.001
Clinical trial			
No (Ref)	1.00		
Yes	1.89	1.11–3.22	0.019
Palliative care			
No (Ref)	1.00		
Yes	1.54	1.37–1.73	<0.001

HR, hazard ratio; CI, confidence interval; Ref, referent category; HSD, high school diploma; ARP, academic/research program; LR, locoregional.

## Discussion

This is the first study utilizing the NCDB to describe nationwide treatment patterns for patients presenting with Stage IVC head and neck cancer, and to determine how treatment functions in the context of other patient demographic and clinical variables in overall survival for patients with distant metastases at presentation. This is also the first multi‐institutional study to report clinical trial enrollment and palliative therapy rates in head and neck cancer patients presenting with distant metastases.

Our study found a combination of locoregional and systemic therapy to be the most common treatment strategy and the one associated with greatest overall survival. While we adjusted for important factors such as comorbidity and age, we were not able to measure the true performance status or metastatic burden of each patient. It is likely that a significant selection bias existed, driving patients with better performance status and minimal metastatic burden to receive more intensive treatment. We also found patients with oropharyngeal primary tumors to have improved overall survival when adjusted for other factors. This may reflect human papilloma virus (HPV)‐positivity, which has been associated with improved prognosis for patients with recurrent/metastatic head and neck cancer 22, 23 and longer recurrence‐free survival 24.

Fourteen patients (0.6%) in our cohort were recorded as being involved in a clinical trial as part of the first course of treatment, which is significantly lower than the nationwide estimate of 2.5% of adult cancer patients 25. The reason for this low rate of participation in clinical trials is likely multifold, and may include the database's narrow definition of clinical trials. Additionally, many trials utilizing radiation exclude patients with distant metastases, and some oncologists may recommend clinical trials only when standard therapies have failed. Investigator preference toward locoregional treatment strategies for patients with lesser disease burden may have also precluded patients from participating in trials including systemic therapies. Several additional reasons for poor accrual in cancer clinical trials have been described in the literature, and include physicians' perception of protocol unavailability, patients' poor performance status and desire for other treatment, distance to the cancer center, and insurance denial 26. In their cross‐sectional analysis of the Clinical Trial Cooperative Group, Murthy et al. described an inverse relationship between age and enrollment in cancer clinical trials, and a significantly lower proportion of Hispanic and Black patients enrolling in clinical trial 27. In surveying 60 noneligible colorectal cancer patients for whether they would participate in a chemotherapeutic trial for colonic adenocarcinoma, Llewellyn‐Thomas and colleagues found that refusers wanted a greater role in decision making and greater treatment benefit 28. A majority of those refusing also cited aversion to randomization as the principal factor in their decision. In light of these factors, the small number of patients in our cohort participating in a clinical trial is unsurprising.

Palliative care refers to therapy administered with the intent to prevent or relieve suffering and to improve quality of life 29, and may be cancer‐directed or primarily involve symptom management. Palliative care plays an important role in head and neck cancer, as quality of life can be influenced by sequelae from both the disease process and subsequent treatment. Implementation of early palliative care has been shown to not only improve quality of life, but to lead to longer survival in select cancer patients 30, 31. The literature shows that head and neck cancer patients receiving more intensive therapeutic regimens are most susceptible to a decline in multiple quality of life measures 32, and that those receiving primary radiotherapy may experience a long‐term decrease in quality of life 33, which may be secondary to dysphagia or xerostomia 34. It has also been shown that pain and diet at 1 year after diagnosis were associated with longer term quality of life measures in head and neck cancer patients 35. End‐of‐life treatment for incurable head and neck cancer patients must be individualized and multifaceted; issues ranging from communication to psychological considerations to treatment venue must be taken into account 36 and managed in a multidisciplinary fashion. Less than 15% of patients in our study were listed as having received palliative cancer‐directed therapy or pain management. This low rate may be due to underreporting, administration of supportive measures by the patient's primary oncologist, or by a medical philosophy that is concerned primarily with eradicating disease and searching for cure, instead of alleviating suffering 37, 38. Unfortunately, the NCDB does not collect data on patient comfort; therefore the adequacy of supportive care delivered to patients in our cohort remains unknown. Those receiving palliative therapies in our study had nonprivate insurance and large lesions, indicating that socioeconomic factors and tumor‐induced dysfunction may play an important role in the decision to administer palliative therapy. Interestingly, we did not find oncologic or clinical variables such as primary site or Charlson score to be associated with the receipt of palliative treatment. In patients presenting with distant metastases, all treatments should be administered with discreet goals of care, patient choice, and functional status in mind. Unfortunately, these factors are not necessarily amenable to a population‐level analysis.

It is important to interpret the findings of this study with its limitations in mind. As with any large administrative dataset, there is potential for incorrect coding, and we aimed to minimize this by cross‐referencing similar variables to maximize consistency. Additionally, due to the availability of certain key variables such as Charlson score and vital status at follow‐up, our analysis is limited to patients diagnosed between years 2003 and 2006. Disease‐free survival is not recorded in the NCDB; therefore we are limited to analyzing overall survival. However, we believe overall survival to be a more than adequate proxy for disease‐free survival in patients with distant metastatic disease, as the vast majority of head and neck cancer patients with distant metastases succumb to their illness within 1 year 39. Due to missing data, it was not possible to determine the relative timing of treatment for the majority of patients or metastatic tumor burden. In addition, p16 status was not coded in the database, which would likely affect survival estimates. Finally, the NCDB likely underestimates the number of patients receiving palliative care, especially those receiving palliative pain management, as the NCDB is unlikely to record a rationale for pain management performed at facilities outside of the participating network 16. The definition of palliative care in a population‐based cohort may also be somewhat nebulous, with many traditionally cancer‐directed treatment modalities such as chemotherapy or radiation used for pain management, as well as symptom and tumor control.

## Conclusion

Patients presenting with distant metastases most commonly had primary tumors of the nasopharynx and hypopharynx, and having a primary tumor of the oropharynx was associated with improved overall survival. A combination of locoregional and systemic therapy was the most common treatment regimen administered, and was the only regimen associated with survival benefit when compared to systemic therapy alone. This association may be due to a selection bias for this treatment approach. A relatively small number of patients were coded as having received palliative care, and very few were enrolled in clinical trials, although these data likely underestimate the true proportions. Obstacles to receiving palliative care should be further explored, as optimizing patient comfort in this setting is of the utmost importance. Efforts aimed at increasing clinical trial accrual should likewise be attempted, as trials represent the frontier of cancer care.

## Conflict of Interest

The authors have no conflicts or disclosures to report.
